# Direct Oral Anticoagulant Therapy for Isolated Distal Deep Vein Thrombosis Associated with Cancer in Routine Clinical Practice

**DOI:** 10.3390/jcm10204648

**Published:** 2021-10-11

**Authors:** Yutaka Ogino, Tomoaki Ishigami, Ryosuke Sato, Hidefumi Nakahashi, Yugo Minamimoto, Yuichiro Kimura, Kozo Okada, Yasushi Matsuzawa, Noriaki Iwahashi, Kiyoshi Hibi, Masami Kosuge, Toshiaki Ebina, Toshiyuki Ishikawa, Kouichi Tamura, Kazuo Kimura

**Affiliations:** 1 Department of Cardiology, Yokohama City University Medical Center, Yokohama 232-0024, Japan; ogino-you@hotmail.co.jp (Y.O.); r_satou@yokohama-cu.ac.jp (R.S.); t146060t@yokohama-cu.ac.jp (H.N.); yugo@yokohama-cu.ac.jp (Y.M.); y_kimura@yokohama-cu.ac.jp (Y.K.); kokada2@yokohama-cu.ac.jp (K.O.); matsu@yokohama-cu.ac.jp (Y.M.); niwahash@urahp.yokohama-cu.ac.jp (N.I.); hibikiyo@urahp.yokohama-cu.ac.jp (K.H.); masami-kosuge@pop06.odn.ne.jp (M.K.); tebina@yokohama-cu.ac.jp (T.E.); c_kimura@yokohama-cu.ac.jp (K.K.); 2 Department of Cardiology, Yokohama City University Hospital, Yokohama 236-0004, Japan; tishika@yokohama-cu.ac.jp (T.I.); tamukou@med.yokohama-cu.ac.jp (K.T.)

**Keywords:** direct oral anticoagulant, isolated distal deep vein thrombosis, cancer

## Abstract

Background: The efficacy and bleeding complications of direct oral anticoagulant (DOAC) therapy for isolated distal deep vein thrombosis (IDDVT) associated with cancer in routine clinical practice remain unclear. Moreover, prior studies on prolonged therapy for IDDVT are limited. Methods: This retrospective study enrolled 1641 consecutive patients with acute venous thromboembolism (VTE) who had received oral anticoagulant therapy, including warfarin or DOAC, between April 2014 and September 2018 in our institutions. In these patients, 200 patients with cancer-associated IDDVT were evaluated. Results: Mean follow-up period was 780 ± 593 days. Major bleeding and VTE recurrence were observed in 22 (11.0%) and 11 (5.5%) patients, respectively. In multivariate analysis, statistically significant factors correlated with major bleeding were advanced cancer stage, high performance status, stomach cancer, and gallbladder cancer; those correlated with all-cause death were advanced cancer stage, high performance status, liver dysfunction, pancreatic cancer, and major bleeding. Cumulative events of major bleeding and recurrence between patients with prolonged DOAC therapy (≥90 days) and those with nonprolonged therapy were not significantly different. Conclusions: Preventing major bleeding is important because it is a significant risk factor for all-cause death. Major bleeding and recurrent events were comparable between prolonged and nonprolonged therapy.

## 1. Introduction

The application of anticoagulant therapy for isolated distal deep vein thrombosis (IDDVT) associated with cancer remains controversial. Some previous studies reported that the high recurrence risk of cancer-associated venous thromboembolism (VTE) and effectiveness of anticoagulant therapy for IDDVT is correlated with active cancer [1,2]. According to these studies, in routine clinical practice, patients with IDDVT associated with active cancer are sometimes prescribed a direct oral anticoagulant (DOAC), especially in the preoperative period. However, in these studies, the examination for the presence of pulmonary embolism (PE) using contrast-enhanced computed tomography (CT) or ventilation–perfusion lung scintigraphy was not routinely performed. There is a paucity of data on the efficacy and bleeding complications of DOAC therapy for “real” IDDVT associated with active cancer in routine clinical practice; moreover, the safety and efficacy of prolonged DOAC therapy (≥90 days) for cancer-associated IDDVT has not been fully elucidated.

This study evaluates bleeding and recurrent complication of patients with cancer-associated IDDVT who received DOAC therapy, and validates the safety and efficacy of prolonged DOAC therapy in routine clinical practice. 

## 2. Materials and Methods

This physician-initiated retrospective study enrolled 1641 consecutive patients with acute VTE who had received oral anticoagulant therapy, including warfarin or DOAC, between April 2014 and September 2018 at Yokohama City University Hospital and Yokohama City University Medical Center. Patient data, including age, sex, VTE etiology, VTE symptomatology, and other VTE-related factors, were collected from hospital charts. VTE diagnosis was based of symptoms and lower-limb findings on ultrasound, contrast-enhanced computed tomography, and ventilation–perfusion lung scintigraphy. For asymptomatic patients, physicians normally diagnose VTE based on the clinical course and objective results on imaging, especially lower-limb ultrasound, which is routinely used in clinical practice [3]. 

In the current study, IDDVT was defined as venous thrombosis of the calf veins, including peroneal, posterior tibial, anterior tibial, and soleus muscle veins below the knee. Major bleeding was defined using the International Society of Thrombosis and Hemostasis criteria: reduction in hemoglobin level by at least 2 g/dL, transfusion of at least 2 units of blood, or symptomatic bleeding in a critical area or organ [4]. In the present study, DOAC withdrawal depended on the treating physician’s judgement. The timing of evaluation for recurrent VTE also depended on the judgment of each physician in the presence of the following symptoms: leg pain, leg swelling, and dyspnea. Regarding asymptomatic cases, increased D-dimer levels were an indication for objective imaging examinations to search for a new thrombus. 

Patients with cancer-associated VTE included those receiving treatment for cancer, such as chemotherapy or radiotherapy; those scheduled to undergo cancer surgery; those with metastasis to other organs; and those with terminal cancer (expected life expectancy of ≤6 months) at the time of diagnosis [5]. We confirmed the specific tumor types, performance status (PS), cancer stage, and performance of chemotherapy at the time of VTE diagnosis. Cancer stage was determined using the TNM classification. Cancers with distal metastasis or the highest malignant grade were classified under stage 4. Liver dysfunction was defined as the presence of a chronic hepatic disease (e.g., cirrhosis) or biochemical evidence of significant hepatic derangement (e.g., bilirubin level of more than twice the upper limit or aspartate aminotransferase/alanine aminotransferase/alkaline phosphatase level of more than thrice the upper limit). This definition was derived from the HAS-BLED score [6]. We evaluated the incidence and characteristics of VTE recurrence, major bleeding, and all-cause death among patients with IDDVT associated with cancer who had undergone DOAC therapy. In the present study, prolonged therapy was defined as anticoagulant therapy with DOAC and/or initial intravenous anticoagulant over 3 months (90 days) according to the guideline of Japanese Circulation Society [7]. We evaluated the relationship between prolonged DOAC therapy, and events of major bleeding and recurrent VTE respectively by conducting a quasi-RCT using the inverse probability of treatment weighting (IPTW) technique [8].

This study was approved by the ethics committee of Yokohama City University Hospital and was conducted in accordance with the Declaration of Helsinki. Written informed consent was obtained from all patients.

### Statistical Analysis

Continuous variables were reported as means ± SDs and categorical variables as frequencies (percentages). Unpaired t-test was used to compare the continuous variables, and the chi-squared test was used to test the difference in the qualitative variables between the groups. For all comparisons, *p* values of <0.05 were considered statistically significant.

In the factors that were significantly associated with major bleeding, recurrent VTE, and all-cause death by using the unpaired t-test or chi-squared test, univariate Cox regression analysis was performed. Thereafter, factors with *p* values of <0.05 were validated in multivariate Cox regression analysis. 

For propensity score calculation, a logistic regression model was used. Multivariable models were adjusted using the IPTW method combined with logistic regression modeling to control for potential confounders: age, sex, body mass index (BMI), the primary site of cancer, cancer stage, performance status, chemotherapy, D-dimer, hemoglobin (Hb), estimated glomerular filtration ration (eGFR), blood platelet count, symptomatic state, use of single drug therapy, use of intravenous anticoagulant, history of stroke, liver dysfunction, diabetes mellitus, and hypertension. We conducted Cox hazards-models analysis to consider survival time of major bleeding and recurrent VTE. Moreover, the Kaplan–Meier method was used to evaluate the cumulative event rate of major bleeding and recurrent VTE.

SPSS ver. 26 (IBM, Armonk, NY, USA) and R 3.3.2 (R Foundation for Statistical Computing, Vienna, Austria) were used for the statistical analysis.

## 3. Results

**Figure 1:**
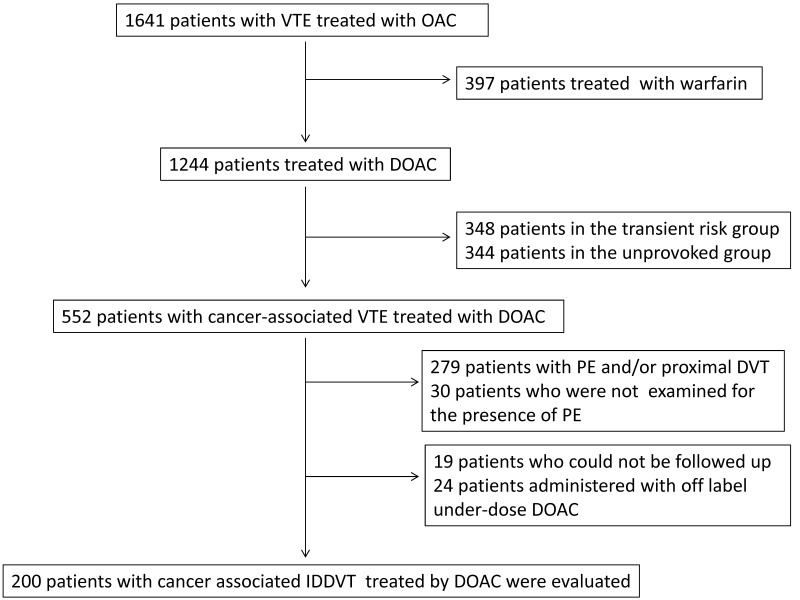
Flowchart of participant inclusion criteria. VTE, venous thromboembolism; OAC, oral anticoagulant; DOAC, direct oral anticoagulant; DVT, deep vein thrombosis; PE, pulmonary embolism; IDDVT, isolated distal deep vein thrombosis.

Between April 2014 and September 2018, 1641 patients with acute VTE were treated with oral anticoagulant therapy at our institutions. Among them, 1244 and 397 patients were treated with DOAC and warfarin, respectively. In the DOAC group, there were 552 patients with cancer-associated VTE; 279 patients had PE and/or proximal DVT and 30 patients were not examined for the presence of PE. Nineteen patients could not be followed-up, and 24 patients received underdose off-label prescription of DOAC. After the exclusion of these patients, 200 patients with cancer-associated IDDVT remained and were evaluated (Figure 1). The mean follow-up period was 780 ± 593 days.

Table 1 shows the characteristics of the 200 patients with cancer-associated IDDVT. Regarding the primary site of cancer, digestive-organ cancers had the greatest number of cases. The number of blood-cancer cases was as follows: malignant lymphoma (*n* = 8) and multiple myeloma (*n* = 2). The number of head and neck cancer cases was as follows: pharyngeal cancer (*n* = 2), oral-cavity cancer (*n* = 2), tongue (*n* = 1), and nasal-cavity cancer (*n* = 1). Regarding treatment in the acute phase, single-drug therapy with 15 mg rivaroxaban BID or 10 mg apixaban BID was used in 6 patients. Intravenous unfractionated heparin was used in 10 patients. 

**Figure 2:**
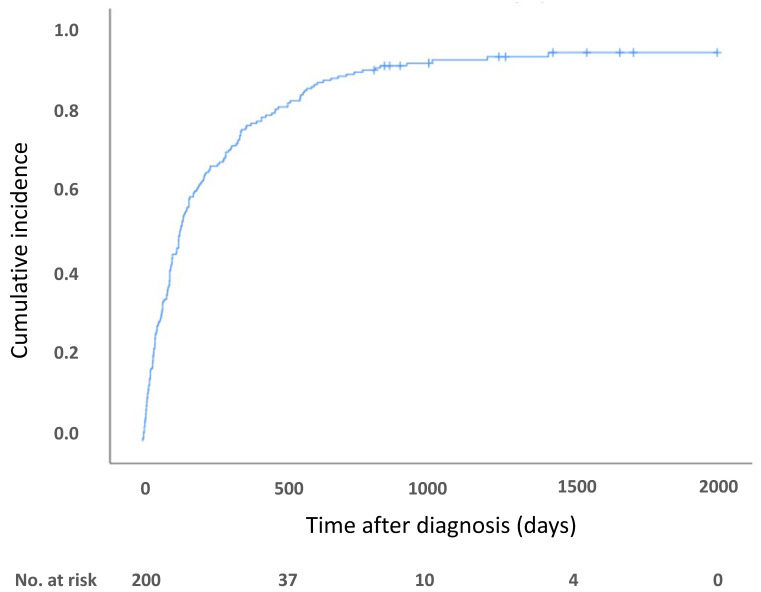
Kaplan–Meier curve-estimated discontinuation of DOAC therapy.

Figure 2 shows the Kaplan–Meier curve estimate of the rate of discontinuation of anticoagulation. In the follow-up period, 187 patients discontinued DOAC; 68, 66, 26, 26, and 1 patient discontinued DOAC because of difficulty of oral intake due to the progression of cancer, physician’s judgement, confirmation of disappearance of thrombus, major and minor bleeding complications, and emergent surgical operation, respectively. The physician’s judgement was that the duration of DOAC was sufficient for their patients without confirmation of disappearance of thrombus. Almost all these patients underwent follow-up lower-limb ultrasound to confirm the reduction of thrombus after discontinuation of DOAC. 

In the follow-up period, 22 (11%) patients developed major bleeding, and 11 (5.5%) patients developed recurrent VTE. A total of 11, 7, 1, 1, 1, and 1 patients had major bleeding in the upper digestive tract, lower digestive tract, brain, biliary tract, intra-abdominal, and aneurysm (rupture), respectively. Fatal bleeding complication under DOAC therapy occurred in one patient (rupture of aneurysm). Among the patients with recurrent VTE, 5, 2, and 4 had nonmassive PE, proximal DVT, and distal DVT, respectively. No patients with recurrent VTE died due to VTE. Table 2 shows the comparison between patients with and without major bleeding. Table 3 shows the comparison between the patients with and without recurrent VTE. Median duration of anticoagulant therapy between the recurrent and nonrecurrent groups was not significant. Table 4 shows the comparison between the patients who survived and those who died. In the present study, 110 patients died; 102, 3, 2, 1, 1, and 1 died from progression of cancer, natural death, aspiration pneumonitis, cerebral infarction, rupture of aorta aneurysm, and unidentified death, respectively.

Table 5 indicates the independent factors correlated with major bleeding, recurrent VTE, and all-cause death after adjustments in Cox regression analysis. In multivariate analysis, advanced cancer stage, high PS, stomach cancer, and gallbladder cancer were correlated with major bleeding. There was no factor correlated with recurrent VTE in this study. Conversely, advanced cancer stage, high PS, pancreatic cancer, liver dysfunction, and major bleeding were independently correlated with all-cause death.

**Figure 3:**
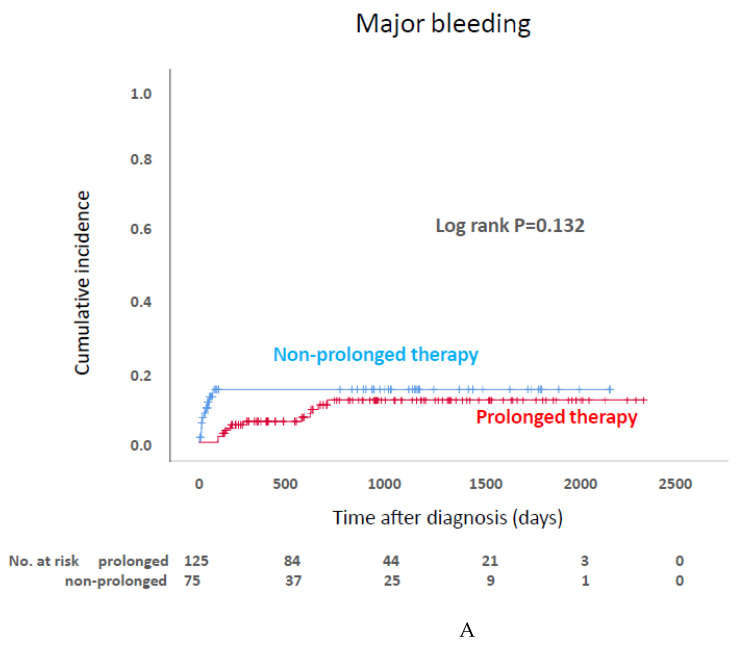

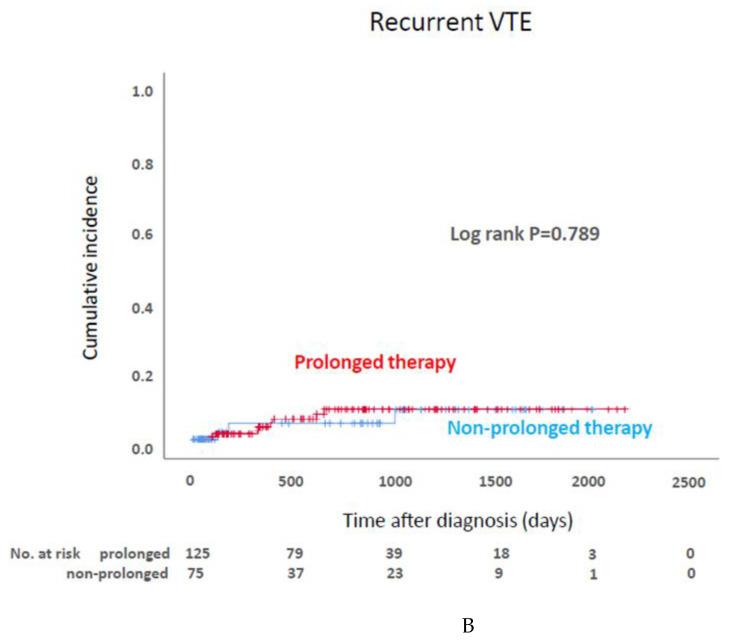
Kaplan–Meier curves estimating incidence of major bleeding (**A**) and recurrent venous thromboembolism (**B**) between patients who received prolonged and nonprolonged therapy.

In the current study, we compared the prolonged-therapy group (*n* = 125) with nonprolonged-therapy group (*n* = 75). Table 6 shows the comparison between the patients with prolonged and nonprolonged therapy. A higher number of patients in the prolonged-therapy group received chemotherapy, which is a significant risk factor for VTE [9]. In the prolonged-therapy group, 44, 34, 23, 11 patients discontinued DOAC because of the difficulty of oral intake due to progression of cancer, physician’s judgement, confirmation of thrombus disappearance, and major and minor bleeding complication. The 13 remaining patients in the prolonged-therapy group continued DOAC therapy in the follow-up period. In the nonprolonged-therapy group, 32, 24, 15, 3, and 1 patients discontinued DOAC because of physician’s judgement, difficulty of oral intake due to the progression of cancer, major and minor bleeding complications, confirmation of thrombus disappearance, and emergent surgical operation. Figure 3 indicates the Kaplan–Meier curve-estimated outcome of major bleeding and recurrent VTE for patients who received prolonged and nonprolonged therapy. The incidences of major bleeding and recurrent VTE after diagnosis were not significantly different between the two groups. Table 7 indicates that the hazard ratio and 95% confidence interval estimated for prolonged therapy correlated with major bleeding, recurrent VTE, and all-cause death. In Cox regression analysis, after propensity matching with IPTW methods, prolonged therapy was not a significant risk factor for major bleeding, recurrent VTE, and all-cause death. 

## 4. Discussion

Prior studies revealed that IDDVT associated with active cancer had a high risk to develop proximal DVT and PE [1,10]. In fact, the American College of Chest Physicians (ACCP) guideline recommends that the management of patients with active cancer should be the same as that for patients with acute proximal DVT [11]. However, due to the lack of routine objective imaging to confirm the presence of PE in some cases, patients included in these studies were not stratified on the basis of whether they had only IDDVT or had both IDDVT and PE, especially asymptomatic PE. Nevertheless, one epidemiological study reported that 29% of distal DVT had concomitant PE [12]. Moreover, in the Italian Master registry, the presence of PE was frequently associated with IDDVT rather than proximal DVT [13]. Our study evaluated the patients who had only IDDVT and were not screened for presence of PE using contrast-enhanced CT or ventilation–perfusion lung scintigraphy. To the best of our knowledge, studies similar to ours are rare.

SELECT-D, Hokusai-VTE cancer, and CARABAGGIO trials, which compared DOAC with dalteparin for cancer-associated VTE, reported the rate of major bleeding of the DOAC group, which ranged approximately from 4.0% to 7.0% [14,15,16]. These randomized control trials (RCTs) included proximal DVT and/or PE and excluded IDDVT. In the current study, even though only IDDVT was included, the rate of major bleeding was higher compared with in these RCTs. In routine clinical practice in Japan, major bleeding events may be higher compared with in Western countries. This may mainly be attributed to the higher incidence of stomach cancer in the current study, as our previous study reported [17]. Regarding stomach cancer, the International Society of Thrombosis suggests LMWH therapy instead of DOACs in patients with gastrointestinal cancer because of their high risk of bleeding [18]. However, LMWH is not permitted to use for the treatment of VTE and is just approved for the prevention of VTE after surgery in Japan. For this reason, in the current study, DOAC therapy was selected even though it was for patients with gastrointestinal cancer. In addition, the low body weight of Japanese people who are affected with cancer might be one reason for the higher rate of major bleeding. For example, the average body weight in the current study was 53.6 kg, compared with 75.7–78.8 kg reported in the Hokusai VTE cancer and CARAVAGGIO trials. Individuals with a higher prevalence of stomach cancer and low body weight, such as Japanese people, should use DOACs cautiously. In this study, gallbladder cancer was also a risk factor for major bleeding. However, the number of gallbladder-cancer cases was too small to ascertain their significance.

Regarding recurrent VTE, there was no fatal PE in the current study. IDDVT has a low risk to develop proximal DVT and PE [19,20]. Even for cancer-associated IDDVT, there is low risk of fatal recurrent VTE under DOAC therapy. Unfortunately, factors correlated with recurrent VTE could not be determined in this study. Further validation on larger numbers of patients is needed to estimate the factors correlated with recurrent VTE.

In Cox regression analysis, major bleeding was an independent risk factor for all-cause death. Among patients with major bleeding, only one had fatal bleeding (rupture of aneurysm). However, major bleeding was associated with prognosis. This might mainly be because therapy for cancer such as chemotherapy or surgery was transiently stopped once major bleeding had developed. The discontinuation of therapy for active cancer might be correlated with shortening of life expectancy. We should pay more attention to avoiding bleeding complications during DOAC therapy for cancer-associated VTE by accordingly adjusting the DOAC dose and the duration of DOAC therapy. 

JCS guidelines recommend anticoagulant therapy for over 3 months (prolonged therapy) for cancer-associated VTE, but prolonged therapy is not recommended for IDDVT [7]. Evidence on the efficacy and safety of prolonged DOAC therapy for IDDVT associated with cancer is scarce, and as such, in routine clinical practice, the duration of DOAC therapy for IDDVT associated with cancer is totally dependent on the judgement of the treating physician. In the current study, physicians tended to select prolonged therapy for patients who receive chemotherapy, which is significant risk factor for VTE. The Kaplan–Meier curve demonstrated the same incidence for recurrent VTE between the prolonged- and nonprolonged-therapy groups in the follow-up period. Patients who develop major bleeding within 90 days stop the anticoagulant within 90 days, and all these patients were included in the nonprolonged-therapy group. For this reason, in the Kaplan–Meier curve estimating major bleeding, the number of major bleeding event within 90 days was larger in the nonprolonged-therapy group compared with the prolonged-therapy group. However, cumulative major bleeding events in the follow-up period were comparable between them. Moreover, prolonged therapy in quasi-RCT was not a significant risk factor for either major bleeding or recurrent VTE in this study. Because the duration of anticoagulant therapy individually varied in this study, the fact that the incidence of major bleeding and recurrence were comparable may have changed in another study design. However, at least in the routine clinical situation where each treating physician decided the duration of DOAC therapy for IDDVT associated with cancer, the incidence of major bleeding and recurrence were comparable by the Kaplan–Meier method in our analysis.

The application of anticoagulant therapy for IDDVT associated with cancer, especially asymptomatic IDDVT, is still controversial. In fact, 151 out of 200 patients (75.5%) had asymptomatic IDDVT in this study. As described in Section 1, previous studies reported that the high recurrence risk of VTE and effectiveness of anticoagulant therapy for IDDVT associated with active cancer, even though IDDVT was not symptomatic. Moreover, Ro et al. estimated that thrombi propagate to the proximal vein from the soleus muscle vein in autopsy cases with massive PE, and reported the importance of preventing soleus vein thrombus [21]. In addition, the ACCP guideline recommends that the management of patients with active cancer should be the same as that for patients with acute proximal DVT. According to these studies and guideline, 200 patients with IDDVT associated with active cancer in the current study were prescribed DOAC. Further prospective studies that research the validity of DOAC therapy for IDDVT associated with cancer are needed.

This study had several limitations. First, both cancer itself, and daily lifestyle factors such as the length of sitting time and drinking behavior affect the formation of IDDVT [22]. The data of lifestyle factors in the current study are limited because it is retrospective. PS, cancer stage, and chemotherapy, which were included in our analysis, might help to to partially predict the daily lifestyle of cancer patients. A prospective study that includes the data of lifestyle factors is needed. Second, this was a retrospective observational study; decisions regarding the validation of recurrent VTE and continuation of anticoagulant therapy depended on the attending physician. This could mainly affect the incidence of recurrent VTE. Third, given that this study only involved two Japanese centers, the number of analyzed patients was relatively small, and most intended patients were Japanese. Fourth, in this study, 158 patients were prescribed edoxaban, 26 rivaroxaban, and 16 were apixaban. Because the numbers of those prescribed rivaroxaban and apixaban were small, we could not evaluate the statistical deference between DOACs. Lastly, compliance to medications was unclear.

## 5. Conclusions

Preventing major bleeding is important because it is a significant risk factor for all-cause death. Major bleeding and recurrent events were comparable between prolonged and nonprolonged therapy.

## Figures and Tables

**Table 1:** Baseline characteristics.

Baseline	*n* = 200
Age (years)	73.2 ± 8.9
Female sex	104 (52.0%)
Body weight (kg)	53.6 ± 11.5
Body mass index	21.6 ± 4.0
Body mass index of ≥30 kg/m^2^	5 (2.5%)
Symptomatic DVT	49 (24.5%)
Primary site of cancer	
Stomach	30 (15.0%)
Colorectum	50 (25.0%)
Pancreas	33 (16.5%)
Esophagus	5 (2.5%)
Bile duct	9 (4.5%)
Gallbladder	4 (2.0%)
Liver	2 (1.0%)
Lung	14 (7.0%)
Breast	5 (2.5%)
Uterus	6 (3.0%)
Ovary	10 (5.0%)
Prostate	3 (1.5%)
Urinary bladder	6 (3.0%)
Kidney	3 (1.5%)
Blood	10 (5.0%)
Head and neck	6 (3.0%)
Nerve	2 (1.0%)
Skin	2 (1.0%)
Stage	
1 to 3	122 (61.0%)
4	78 (39.0%)
Performance status	
0	112 (56.0%)
1	73 (36.5%)
2	14 (7.0%)
3 to 4	1 (0.5%)
Chemotherapy	115 (57.5%)
Hypertension	78 (39.0%)
Diabetes mellitus	30 (15.0%)
Previous stroke	6 (3.0%)
Liver dysfunction	16 (8.0%)
Laboratory results at diagnosis	
D-dimer (µg/mL)	8.6 ± 14.6
eGFR (mL/min/1.73 m^2^)	70.2 ± 20.3
Hemoglobin (g/dL)	11.6 ± 1.80
Platelet (×10^4^/dL)	23.6 ± 9.8
Treatment in the acute phase	
Single-drug therapy	6 (3.0%)
Intravenous anticoagulant	10 (5.0%)
Drug at diagnosis	
Antiplatelet agents	8 (4.0%)
Nonsteroidal anti-inflammatory drugs	29 (14.5%)
Corticosteroids	13 (6.5%)

DVT, deep vein thrombosis; eGFR, estimated glomerular filtration ration.

**Table 2:** Comparison between patients with bleeding and without bleeding.

	Bleeding (*n* = 22)	No Bleeding (*n* = 178)	*p* Value
Age (years)	74.5 ± 8.8	73.0 ± 8.9	0.47
Female sex	10 (45.5%)	94 (52.8%)	0.51
Body weight (kg)	49.5 ± 8.5	54.1 ± 11.7	0.079
Body mass index	20.6 ± 3.4	21.8 ± 4.0	0.18
Body mass index of ≥30 kg/m^2^	0 (0.0%)	5 (2.8%)	0.43
Symptomatic DVT	10 (45.5%)	39 (21.9%)	0.015
Primary site of cancer			
Stomach	8 (36.4%)	22 (12.4%)	0.0029
Colorectum	4 (18.2%)	46 (25.8%)	0.43
Pancreas	4 (18.2%)	29 (16.3%)	0.82
Esophagus	0 (0.0%)	5 (2.8%)	0.43
Bile duct	0 (0.0%)	9 (5.1%)	0.28
Gallbladder	2 (9.1%)	2 (1.1%)	0.012
Liver	0 (0.0%)	2 (1.1%)	0.62
Lung	1 (4.5%)	13 (7.3%)	0.63
Breast	1 (4.5%)	4 (2.2%)	0.51
Uterus	0 (0.0%)	6 (3.4%)	0.38
Ovary	2 (9.1%)	8 (4.5%)	0.35
Prostate	0 (0.0%)	3 (1.7%)	0.54
Urinary bladder	0 (0.0%)	6 (3.4%)	0.38
Kidney	0 (0.0%)	3 (1.7%)	0.54
Blood	0 (0.0%)	10 (5.6%)	0.25
Head and neck	0 (0.0%)	6 (3.4%)	0.38
Nerve	0 (0.0%)	2 (1.1%)	0.62
Skin	0 (0.0%)	2 (1.1%)	0.62
Stage			
1 to 3	8 (36.4%)	114 (64.0%)	
4	14 (63.6%)	64 (36.0%)	0.012
Performance status			
0	4 (18.2%)	108 (60.7%)	<0.001
1	14 (63.6%)	59 (33.1%)	0.005
2 to 4	4 (18.2%)	11 (6.2%)	0.044
Chemotherapy	16 (72.3%)	99 (55.6%)	0.13
Hypertension	12 (54.5%)	66 (37.1%)	0.11
Diabetes mellitus	5 (22.7%)	25 (14.0%)	0.28
Previous stroke	1 (4.5%)	5 (2.8%)	0.65
Liver dysfunction	1 (4.5%)	15 (8.4%)	0.53
Laboratory results at diagnosis			
D-dimer (µg/mL)	5.75 ± 5.2	8.96 ± 15.3	0.38
eGFR (mL/min/1.73 m^2^)	67.8 ± 26.3	70.5 ± 19.5	0.57
Hemoglobin (g/dL)	11.2 ± 2.1	11.6 ± 1.8	0.30
Platelet (×10^4^/dL)	26.8 ± 9.3	23.2 ± 9.8	0.11
Treatment in the acute phase			
Single-drug therapy	1 (4.5%)	5 (2.8%)	0.65
Intravenous anticoagulant	1 (4.5%)	9 (5.1%)	0.92
Drug at diagnosis			
Antiplatelet agents	1 (4.5%)	7 (3.9%)	0.89
Nonsteroidal anti-inflammatory drugs	3 (13.6%)	26 (14.6%)	0.90
Corticosteroids	0 (0.0%)	13 (7.3%)	0.19

DVT, deep vein thrombosis.

**Table 3:** Comparison between patients with and without bleeding.

	Recurrence (*n* = 11)	No Recurrence (*n* = 189)	*p* Value
Age (years)	69.5 ± 9.3	73.4 ± 8.9	0.16
Female sex	5 (45.5%)	99 (52.4%)	0.65
Body weight (kg)	56.3 ± 10.3	53.4 ± 11.6	0.42
Body mass index	22.3 ± 2.57	21.6 ± 4.0	0.56
Body mass index ≥ 30 kg/m^2^	0 (0.0%)	5 (2.6%)	0.58
Symptomatic DVT	4 (36.3%)	45 (23.8%)	0.35
Primary site of cancer			
Stomach	2 (18.2%)	28 (14.8%)	0.76
Colorectum	4 (36.4%)	46 (24.3%)	0.37
Pancreas	1 (9.1%)	32 (16.9%)	0.50
Esophagus	0 (0.0%)	5 (2.6%)	0.58
Bile duct	0 (0.0%)	9 (4.8%)	0.46
Gallbladder	0 (0.0%)	4 (2.1%)	0.63
Liver	0 (0.0%)	2 (1.1%)	0.73
Lung	1 (9.1%)	13 (6.9%)	0.78
Breast	0 (0.0%)	5 (2.6%)	0.58
Uterus	0 (0.0%)	6 (3.2%)	0.55
Ovary	1 (9.1%)	9 (4.8%)	0.52
Prostate	0 (0.0%)	3 (1.6%)	0.67
Urinary bladder	1 (9.1%)	5 (2.6%)	0.22
Kidney	0 (0.0%)	3 (1.6%)	0.67
Blood	0 (0.0%)	10 (5.3%)	0.43
Head and neck	0 (0.0%)	6 (3.4%)	0.55
Nerve	1 (9.1%)	1 (0.53%)	0.006
Skin	0 (0.0%)	2 (1.1%)	0.73
Stage			
1 to 3	8 (72.7%)	114 (60.3%)	
4	3 (27.3%)	75 (39.7%)	0.41
Performance status			
0	9 (81.8%)	103 (54.5%)	0.076
1	2 (18.2%)	71 (37.6%)	0.19
2 to 4	0 (0.0%)	15 (7.9%)	0.33
Chemotherapy	6 (54.5%)	109 (57.7%)	0.84
Hypertension	6 (54.5%)	72 (38.1%)	0.28
Diabetes mellitus	2 (18.2%)	28 (14.8%)	0.76
Previous stroke	1 (9.1%)	5 (2.6%)	0.22
Liver dysfunction	1 (9.1%)	15 (7.9%)	0.89
Laboratory results at diagnosis			
D-dimer (µg/mL)	4.7 ± 4.2	8.9 ± 15.1	0.36
eGFR (mL/min/1.73 m^2^)	70.5 ± 23.0	70.1 ± 20.1	0.96
Hemoglobin (g/dL)	12.5 ± 1.6	11.5 ± 1.8	0.071
Platelet (×10^4^/dL)	24.9 ± 10.3	23.5 ± 9.8	0.64
Treatment in the acute phase			
Single-drug therapy	0 (0.0%)	6 (3.4%)	0.55
Intravenous anticoagulant	0 (0.0%)	10 (5.3%)	0.43
Drug at diagnosis			
Antiplatelet agents	1 (9.0%)	7 (3.7%)	0.38
Nonsteroidal anti-inflammatory drugs	2 (18.2%)	27 (14.3%)	0.72
Corticosteroids	0 (0.0%)	13 (6.9%)	0.37
Median duration of anticoagulant (days)	163	126	0.40

DVT, deep vein thrombosis.

**Table 4:** Comparison between patients with and without death.

	Death (*n* = 110)	No Death (*n* = 90)	*p* Value
Age (years)	73.0 ± 9.4	73.4 ± 8.3	0.73
Female sex	58 (52.7%)	46 (51.1%)	0.82
Body weight (kg)	52.8 ± 11.9	54.5 ± 10.9	0.31
Body mass index	21.2 ± 4.2	22.1 ± 3.4	0.13
Body mass index ≥ 30 kg/m^2^	1 (0.91%)	4 (4.4%)	0.11
Symptomatic DVT	26 (23.6%)	23 (25.6%)	0.75
Primary site of cancer			
Stomach	18 (16.4%)	12 (13.3%)	0.55
Colorectum	24 (21.8%)	26 (28.9%)	0.25
Pancreas	27 (24.5%)	6 (6.7%)	<0.001
Esophagus	4 (3.6%)	1 (1.1%)	0.26
Bile duct	4 (3.6%)	5 (5.6%)	0.51
Gallbladder	4 (3.6%)	0 (0.0%)	0.068
Liver	0 (0.0%)	2 (2.2%)	0.12
Lung	7 (6.4%)	7 (7.8%)	0.70
Breast	2 (1.8%)	3 (3.3%)	0.49
Uterus	2 (1.8%)	4 (4.4%)	0.28
Ovary	4 (3.6%)	6 (6.7%)	0.33
Prostate	2 (1.8%)	1 (1.1%)	0.68
Urinary bladder	2 (1.8%)	4 (4.4%)	0.28
Kidney	0 (0.0%)	3 (3.3%)	0.054
Blood	3 (2.7%)	7 (7.8%)	0.10
Head and neck	5 (4.5%)	1 (1.1%)	0.16
Nerve	0 (0.0%)	2 (2.2%)	0.12
Skin	2 (1.8%)	0 (0.0%)	0.20
Stage			
1 to 3	39 (35.5%)	83 (92.2%)	
4	71 (64.5%)	7 (7.8%)	<0.001
Performance status			
0	35 (31.8%)	77 (85.6%)	<0.001
1	60 (54.5%)	13 (14.4%)	<0.001
2 to 4	15 (13.6%)	0 (0.0%)	<0.001
Chemotherapy	81 (73.6%)	34 (37.8%)	<0.001
Hypertension	40 (36.3%)	38 (42.2%)	0.40
Diabetes mellitus	19 (17.3%)	11 (12.2%)	0.32
Previous stroke	4 (3.6%)	2 (2.2%)	0.56
Liver dysfunction	15 (16.7%)	1 (1.1%)	0.001
Laboratory results at diagnosis			
D-dimer (µg/mL)	10.8 ± 14.9	6.16 ± 14.1	0.034
eGFR (mL/min/1.73 m^2^)	72.6 ± 21.1	67.2 ± 18.9	0.063
Hemoglobin (g/dL)	11.1 ± 1.7	12.1 ± 1.8	<0.001
Platelet (×10^4^/dL)	24.3 ± 9.6	22.7 ± 10.1	0.26
Treatment in the acute phase			
Single-drug therapy	3 (2.7%)	3 (3.3%)	0.80
Intravenous anticoagulant	6 (5.5%)	4 (4.4%)	0.74
Drug at diagnosis			
Antiplatelet agents	5 (4.5%)	3 (3.3%)	0.66
Nonsteroidal anti-inflammatory drugs	15 (13.6%)	14 (15.6%)	0.70
Corticosteroids	6 (5.5%)	7 (7.8%)	0.51
Major bleeding	20 (18.2%)	2 (2.2%)	<0.001
Recurrent VTE	4 (3.6%)	7 (7.8%)	0.20

DVT, deep vein thrombosis; VTE, venous thromboembolism.

**Table 5:** Factors correlated with major bleeding, recurrent VTE, and all-cause death.

	Univariate HR (95% CI)	*p* Value	Multivariate HR (95% CI)	*p*-Value
Major bleeding				
Symptomatic DVT	2.559 (1.105–5.924)	0.028	2.351 (0.988–5.597)	0.053
Stomach cancer	3.386 (1.417–8.091)	0.006	2.749 (1.050–7.202)	0.040
Gallbladder	15.33 (3.404–69.06)	<0.001	11.86 (2.196–64.00)	0.004
Stage 4	4.854 (1.996–11.80)	<0.001	2.686 (1.036–6.964)	0.042
Performance status	2.850 (1.797–4.518)	<0.001	2.667 (1.528–4.656)	0.001
Recurrent VTE				
Nerve	6.024 (0.765–47.46)	0.088		
All-cause death				
Pancreatic cancer	2.460 (1.587–3.811)	<0.001	1.912 (1.182–3.093)	0.008
Stage 4	7.415 (4.901–11.22)	<0.001	3.712 (2.289–6.021)	<0.001
Performance status	2.359 (1.903–2.924)	<0.001	1.860 (1.345–2.573)	<0.001
Chemotherapy	2.771 (1.809–4.246)	<0.001	1.222 (0.749–1.996)	0.422
Liver dysfunction	4.568 (2.623–7.956)	<0.001	2.513 (1.207–5.236)	0.014
D-dimer	1.013 (1.004–1.022)	0.004	1.007 (0.993–1.022)	0.321
Hemoglobin level	0.823 (0.740–0.916)	<0.001	0.893 (0.791–1.008)	0.066
Major bleeding	3.067 (1.867–5.040)	<0.001	2.149 (1.200–3.851)	0.010

VTE, venous thromboembolism; DVT, deep vein thrombosis, HR, hazard ratio; CI, confidential interval.

**Table 6:** Comparison between patients with prolonged and nonprolonged therapy.

	Prolonged Therapy (*n* = 125)	Nonprolonged Therapy (*n* = 75)	*p* Value
Age (years)	73.4 ± 9.2	72.8 ± 8.4	0.62
Female sex	59 (47.2%)	45 (60%)	0.079
Body weight (kg)	55.0 ± 11.6	51.2 ± 11.0	0.028
Body mass index	22.1 ± 4.10.	20.9 ± 3.68	0.045
Body mass index ≥ 30 kg/m^2^	3 (2.4%)	2 (2.7%)	0.91
Symptomatic DVT	36 (28.8%)	13 (17.3%)	0.068
Primary site of cancer			
Stomach	21 (16.8%)	9 (12.0%)	0.36
Colorectum	28 (22.4%)	22 (29.3%)	0.27
Pancreas	20 (16.0%)	13 (17.3%)	0.81
Esophagus	2 (1.6%)	3 (4.0%)	0.29
Bile duct	3 (2.4%)	6 (8.0%)	0.064
Gallbladder	1 (0.8%)	3 (4.0%)	0.12
Liver	1 (0.8%)	1 (1.3%)	0.71
Lung	10 (8.0%)	4 (5.3%)	0.47
Breast	4 (3.2%)	1 (1.3%)	0.41
Uterus	5 (4.0%)	1 (1.3%)	0.28
Ovary	8 (6.4%)	2 (2.7%)	0.24
Prostate	1 (0.8%)	2 (2.7%)	0.29
Urinary bladder	6 (4.8%)	0 (0.0%)	0.054
Kidney	3 (2.4%)	0 (0.0%)	0.18
Blood	6 (4.8%)	4 (5.3%)	0.87
Head and neck	5 (4.0%)	1 (1.3%)	0.28
Nerve	0 (0.0%)	2 (2.7%)	0.067
Skin	1 (0.8%)	1 (1.3%)	0.71
Stage			
1 to 3	78 (62.4%)	44 (58.7%)	
4	47 (37.6%)	31 (41.3%)	0.60
Performance status			
0	73 (58.4%)	39 (52.0%)	0.38
1	46 (36.8%)	27 (36.0%)	0.91
2 to 4	6 (4.8%)	9 (12.0%)	0.061
Chemotherapy	80 (64.0%)	35 (46.7%)	0.016
Hypertension	50 (40.0%)	28 (37.3%)	0.71
Diabetes mellitus	21 (16.8%)	9 (12.0%)	0.36
Previous stroke	3 (2.4%)	3 (4.0%)	0.52
Liver dysfunction	6 (4.8%)	10 (13.3%)	0.031
Laboratory results at diagnosis			
D-dimer (µg/mL)	7.55 ± 13.5	10.5 ± 16.3	0.20
eGFR (mL/min/1.73 m^2^)	70.6 ± 18.4	69.5 ± 23.2	0.70
Hemoglobin (g/dL)	11.6 ± 1.70	11.4 ± 1.92	0.42
Platelet (×10^4^/dL)	23.9 ± 10.0	23.2 ± 9.5	0.62
Treatment in the acute phase			
Single-drug therapy	6 (4.8%)	0 (0.0%)	0.054
Intravenous anticoagulant	9 (7.2%)	1 (1.3%)	0.065
Drug at diagnosis			
Antiplatelet agents	6 (4.8%)	2 (2.7%)	0.46
Nonsteroidal anti-inflammatory drugs	18 (14.4%)	11 (14.7%)	0.96
Corticosteroids	9 (7.2%)	4 (5.3%)	0.60
Median duration of anticoagulant (days)	281	35	<0.01

DVT, deep vein thrombosis.

**Table 7:** Validation of prolonged therapy for major bleeding, recurrent VTE, and all-cause death after propensity matching with IPTW method.

	Hazard Ratio	95% CI	*p*-Value
Major bleeding	0.62	0.207–1.884	0.40
Recurrent VTE	2.01	0.423–9.50	0.40
All-cause death	0.99	0.512–1.930	0.98

VTE, venous thromboembolism; IPTW, inverse probability of treatment weighting; CI, confidential interval.

## Data Availability

Deidentified participant data will not be shared.
